# Cell-type-specific m^1^A dynamics are associated with microglial phenotypic transition and neuronal metabolic adaptation during spinal cord injury

**DOI:** 10.1371/journal.pcbi.1014573

**Published:** 2026-07-24

**Authors:** Chi Zhang, Shaolong Li, Ruizhi Jiang, Heng Duan, Chuang Li, Enlin Qi, Mingxin Wu, Xueying Li, Shiqing Feng, Hengxing Zhou

**Affiliations:** 1 Department of Orthopaedics, The Second Qilu Hospital of Shandong University, Shandong University Centre for Orthopaedics, Cheeloo College of Medicine, Shandong University, Jinan, China; 2 Department of Orthopaedics, Qilu Hospital of Shandong University, Shandong University Centre for Orthopaedics, Advanced Medical Research Institute, Cheeloo College of Medicine, Shandong University, Jinan, China; 3 Department of Orthopaedics, Tianjin Medical University General Hospital, International Science and Technology Cooperation Base of Spinal Cord Injury, Tianjin Key Laboratory of Spine and Spinal Cord, Tianjin, China; 4 Shandong University Centre for Orthopaedics, Advanced Medical Research Institute, Cheeloo College of Medicine, Shandong University, Jinan, China; University of Wisconsin Madison, UNITED STATES OF AMERICA

## Abstract

m^1^A (N1-methyladenosine) is an important epigenetic mechanism that regulates the onset and progression of many diseases, including spinal cord injury (SCI). To investigate the overall changes in m^1^A following SCI, we analyzed transcriptomic sequencing data from SCI samples and assigned m^1^A scores based on the levels of m^1^A regulatory factors. In this study, the m^1^A score is an inferred proxy calculated from the expression of m^1^A regulator genes (writers/erasers/readers). It does not directly measure RNA m^1^A modification levels. Our results show that the m^1^A score increased within the first day after SCI and then decreased, falling below baseline by day 3 and day 7. Further analysis revealed that microglia and neurons are the two cell types with the most significant changes in the m^1^A score. In microglia, m^1^A score decreased at all time points, whereas in neurons, m^1^A score increased at all time points. Additionally, pseudotime and functional enrichment analyses suggested that the m^1^A score is associated with microglial phenotypic transition and neuronal energy metabolism, which was further validated by both in vivo and in vitro experiments. In summary, our study unveils the characteristic changes of m^1^A at both the bulk and single-cell levels following SCI, and suggests potential links to neuronal function and supports the rationale for further studies exploring m^1^A-related regulators as therapeutic targets in SCI.

## Author summary

Spinal cord injury (SCI) is a severe neurological condition that often results in long-term disability, yet the molecular mechanisms driving injury progression and recovery remain incompletely understood. Increasing evidence suggests that epigenetic regulation, including RNA modifications, plays an important role in shaping cellular responses after injury. N1-methyladenosine (m^1^A) is one such RNA modification implicated in multiple diseases, but its dynamic changes following SCI are unclear. In this study, we analyzed transcriptomic data from SCI samples and inferred an m^1^A score based on the expression of known m^1^A regulatory genes (writers, erasers, and readers). This score represents an indirect proxy and does not directly measure RNA m^1^A modification levels. We observed a rapid increase in the m^1^A score within the first day after injury, followed by a decline below baseline at later time points. At the single-cell level, microglia showed consistently decreased m^1^A scores, whereas neurons displayed sustained increases. Further analyses suggest that these changes may relate to microglial phenotypic transition and neuronal energy metabolism. Our findings provide insight into m^1^A-associated regulation after SCI and highlight m^1^A-related regulators as potential therapeutic targets.

## 1. Introduction

Spinal cord injury (SCI) is a severe disabling injury, often leading to the loss of sensory, motor, and autonomic functions below the level of the injury [1]. SCI can be classified into traumatic spinal cord injury and non-traumatic spinal cord injury [2]. Traumatic SCI is often caused by motor vehicle collisions, falls, and violent injuries [3], whereas non-traumatic SCI is commonly due to tumors, infections, and degenerative spinal diseases [4]. A recent global burden of disease study on SCI indicated that as of 2019, there were 20.6 million SCI patients worldwide, with an annual incidence of 909,000 cases. East Asia reported the highest annual incidence, with 236,000 new cases [5]. Despite efforts in SCI treatment, there is currently no evidence to support the efficacy of drugs or surgery in acute SCI [6–8]. Therefore, understanding the pathophysiological mechanisms of SCI is pivotal for treatments.

Neurons are the most crucial cell type in the spinal cord, and the unsatisfactory therapeutic outcomes in SCI are closely related to the poorly regenerative ability of neurons [9]. Primary injury can lead to direct neuronal death [3], while secondary neuronal damage is often attributed to oxidative stress [10]. The extent and severity of neuronal damage directly impact the prognosis of SCI [11]. Studies have shown that m^1^A methylation on tRNA is closely associated with early oxidative stress damage [12]. However, whether neuronal oxidative stress injury following SCI is also linked to methylation remains unproven.

Microglia are also critical in the early stages of spinal cord injury. Under non-injury conditions, microglia tend to exhibit an M2 phenotype [13], but after SCI, the inflammatory environment leads microglia to polarize toward the M1 phenotype [14]. M1 microglia are beneficial for clearing cell debris [15] but also exacerbate neuroinflammation [16], which is detrimental to neuronal survival. Recent studies have further subdivided microglia into five subgroups based on characteristic cell markers: homeostatic, inflammatory, dividing, migrating, and interferon microglia [17], offering a more detailed understanding of microglial functions and molecular characteristics. Research has shown that epigenetic regulation, including m^6^A, can modulate microglial inflammatory responses [18–20]. However, there is limited research on m^1^A modifications in microglia, despite their similar importance.

The m^1^A (N1-methyladenosine) modification involves the addition of a methyl group to the N1 position of adenosine by m^1^A methyltransferase. This modification was first discovered in 1961 by Dunn et al. [21]. m^1^A levels are modulated by m^1^A regulatory factors, including methyltransferases (writers), demethylases (erasers), and m^1^A-binding reader proteins [22,23]. Early studies suggested that m^1^A was primarily present in tRNA [24], with fewer reports in mRNA. However, with the development of MeRIP, m^1^A modification in mRNA has been gradually identified [25]. The m^1^A modification of mRNA may be related to promoting translation, but its exact function remains unclear [25]. Among tRNAs, five m^1^A modification sites have been identified, located at positions 9, 14, 22, 57, and 58 [26]. Methylation at position 9 is mainly catalyzed by TRMT10C [27], whereas methylation at position 58 is primarily mediated by the TRMT6-TRMT61A complex [28]. In mRNA, m^1^A modifications are also largely installed by TRMT10C and TRMT6 [26,29]. Demethylation of m^1^A is mainly carried out by the ALKBH family proteins and FTO. Specifically, ALKBH3 is responsible for the demethylation of both mRNA and tRNA [30,31], whereas ALKBH1 and FTO have so far only been observed to exert demethylation activity on tRNA [32,33]. The major m^1^A reader proteins belong to the YTH family, including YTHDF1/2/3 and YTHDC1. These proteins regulate RNA splicing, nuclear export, translation, and degradation by binding to methylated RNA [34, 35]. Although YTH proteins were initially considered primarily as m^6^A readers, recent studies have demonstrated that they are also capable of recognizing m^1^A modifications [36]. m^1^A has been implicated in the pathogenesis of various diseases, including cancer cell metabolism [25], abdominal aortic aneurysm [37], and melanoma [38]. However, research on m^1^A in the nervous system is sparse, with most studies focusing on gliomas [39,40]. A few studies have confirmed the relationship between m^1^A and neurodegenerative disorders: reduced ALKBH3 expression leads to a significant increase in overall m^1^A levels, and m^1^A modifications in mRNA with CAG repeat sequences contribute to neurodegeneration in vivo [41]. There is almost no reported research on the impact of m^1^A on SCI. Therefore, we aim to investigate the changes in m^1^A in different cell types before and after SCI. Directly measuring tissue or cell methylation levels is challenging, so we used UCell to compute an m^1^A score based on the expression of m^1^A regulators (writers/erasers/readers), which serves as a proxy for m^1^A-related regulatory activity and not a direct measurement of RNA m^1^A modification levels. We further mapped the m^1^A score across different cell types before and after SCI to explore its distribution and the potential biological functions with which it may be associated. Our results revealed that m^1^A score changes significantly in microglia and neurons and may be involved in the phenotypic transition of microglia and energy metabolism in neurons following SCI.

## 2. Results

### 2.1. The dynamic changes of m^1^A score at the bulk level post-SCI and its significant correlation with energy metabolism pathways

**Fig 1:**
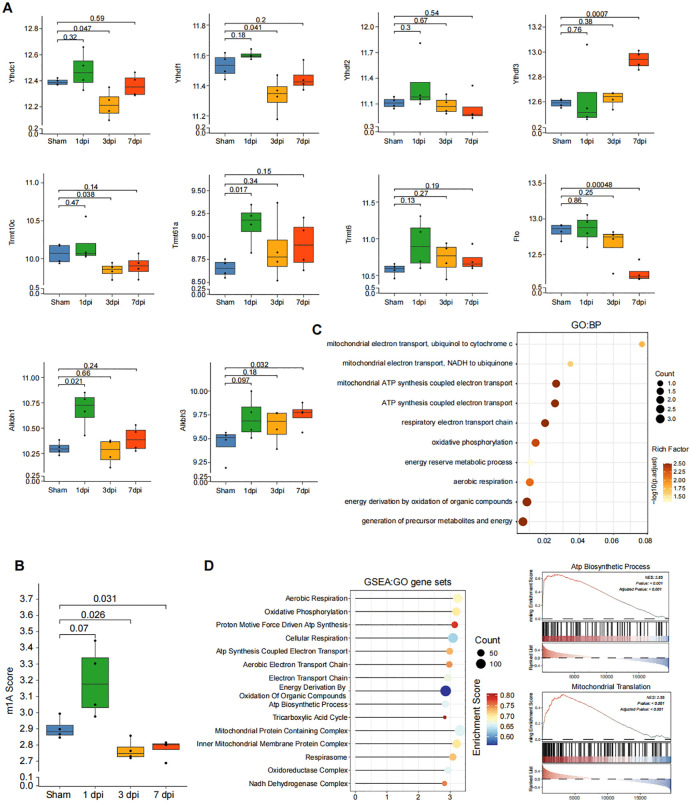
m^1^A regulatory factors and m^1^A score at the bulk level after spinal cord injury. (A) Expression changes of m^1^A regulatory factors (*Ythdc1, Ythdf1, Ythdf2, Ythdf3, Trmt10c, Trmt61a, Trmt6, Alkbh3, Alkbh1, Fto*) at the bulk level after spinal cord injury. All expression changes are presented for the SCI group relative to the sham baseline. Each dot represents one independent tissue sample (sham: n = 3 mice; SCI: n = 3 mice). Exact *P* values are provided in the figure; *P* < 0.05 was considered statistically significant; (B) Changes in the m^1^A score at the bulk level after spinal cord injury. The m^1^A score in the SCI group was compared with that in the sham group, which served as the baseline control. Each dot represents one independent tissue sample (sham: n = 3; SCI: n = 3). Exact *P* values are provided in the figure; *P* < 0.05 was considered statistically significant; (C) GO enrichment analysis showing that m^1^A regulatory factors functions are mainly associated with the mitochondrial electron transport chain; (D) GSEA showing that m^1^A score-related gene functions are primarily involved in energy metabolism and mitochondrial function.

To investigate the changes in m^1^A modification characteristics after SCI, we selected 10 previously reported m^1^A regulatory factors for further analysis (*Ythdc1, Ythdf1, Ythdf2, Ythdf3, Trmt10c, Trmt61a, Trmt6, Alkbh3, Alkbh1, Fto*) [26–36]. First, we used bulk RNA-seq data at different time points after SCI (Sham: uninjured group; 1 dpi: one day post-injury; 3 dpi: three days post-injury; 7 dpi: seven days post-injury) to analyze the overall m^1^A regulatory factor levels (Fig 1A). We also applied the GSVA method to score the overall m^1^A regulatory factors and calculated the m^1^A score to reflect the trends of m^1^A regulatory factor changes over time. The results showed that, except for *Ythdf3*, other m^1^A regulatory factors and m^1^A score exhibited nearly identical patterns after SCI: an increase in the acute phase (1 dpi) followed by a decrease in the subacute phase (3 dpi, 7 dpi) (Fig 1A and 1B). This suggests that m^1^A regulatory factors may play different roles at different stages of SCI. Functional enrichment analysis of m^1^A regulatory factors and m^1^A score revealed that m^1^A regulatory factors are primarily associated with the mitochondrial respiratory chain (Fig 1C), while m^1^A score is mainly enriched in ATP energy metabolism and mitochondrial function-related pathways (Fig 1D). We hypothesize that the disruption of energy metabolism in the subacute phase of SCI may be closely associated with the lower m^1^A scores.

### 2.2. Cellular heterogeneity of m^1^A score changes at the single-cell level post-SCI

**Fig 2:**
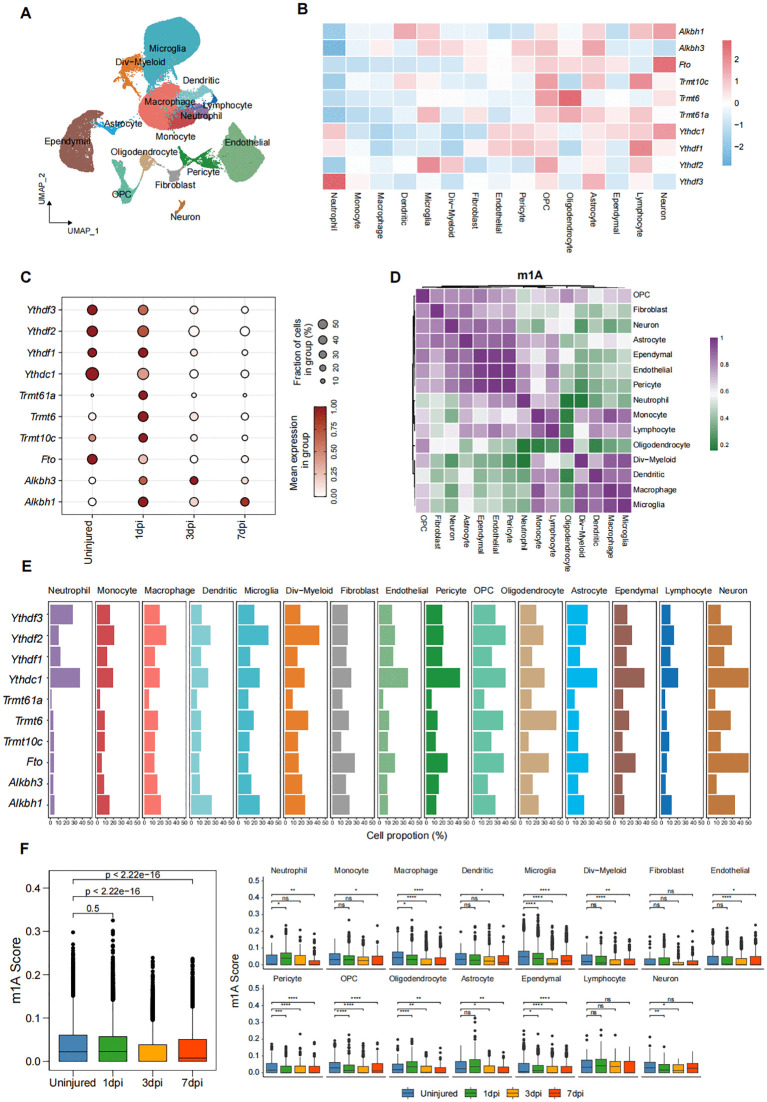
m^1^A regulatory factors and m^1^A score at the single-cell level after spinal cord injury. (A) UMAP plot showing the 15 major cell types from spinal cord tissue, with each dot representing a single cell; (B) Differential expression of key m^1^A regulatory factors across different cell types. Expression patterns and fold-change directions were interpreted relative to the Uninjured baseline when comparisons involved post-injury groups. Color changes representing expression levels (red indicates higher expression, blue indicates lower expression); (C) Overall expression of each m^1^A regulatory factor at different time points after spinal cord injury, with dot size representing the proportion of cells in the group and color indicating average expression, with deeper colors indicating higher expression; (D) Correlation heatmap of the expression levels of m^1^A regulatory factors across different cell types, with color intensity representing the level of correlation (*r* > 0: positive correlation, *r* < 0: negative correlation); (E) Expression distribution of each m^1^A regulatory factor across different cell types; (F) Changes in m^1^A scores at the overall and individual cell type levels at different injury time points. For all post-injury comparisons, the Uninjured group served as the baseline control, and score changes were interpreted relative to this baseline. Uninjured: n = 3 mice; SCI: n = 2 mice. Each dot represents one cell. Significance levels are defined as follows: *P* < 0.05 (*), *P* < 0.01 (**), *P* < 0.001 (***), and *P* < 0.0001 (****).

We performed additional analysis using single-cell sequencing data. To ensure consistency with the established cellular annotation, we referenced the UMAP spatial coordinates and cell classification from the corresponding published studies during clustering. After filtering, cells were clustered into 15 groups: neurons, lymphocytes, ependymal cells, astrocytes, oligodendrocytes, oligodendrocyte precursor cells, pericytes, endothelial cells, fibroblasts, dividing myeloid cells, microglia, dendritic cells, macrophages, monocytes, and neutrophils (Fig 2A). Overall, after SCI, m^1^A regulatory factors were mainly upregulated in neurons, lymphocytes, astrocytes, and oligodendrocyte precursor cells; they were downregulated in neutrophils, monocytes, and macrophages, with more heterogeneous expression patterns observed in microglia and fibroblasts (Fig 2B), indicating cell-specific expression of m^1^A regulatory factors. Further analysis of the level of m^1^A regulatory factors at different phases post-SCI showed an overall increase at 1 dpi, followed by a decrease at 3 dpi and 7 dpi (Fig 2C), which confirms the time-specific expression of m^1^A regulatory factors after SCI. Due to the cell-type-specific expression of m^1^A regulatory factors, different cell types could be distinguished based on m^1^A regulatory factor expression (Fig 2D). Further analysis of the distribution of different m^1^A regulatory factors in each cell type revealed that *Ythdf1, Ythdf2, Trmt6*, and *Fto* were higher in most cell types (Fig 2E). The differences in m^1^A regulatory factor expression across cell types suggest their involvement in distinct cellular functions. Finally, we analyzed the temporal changes of m^1^A score in the 15 cell subgroups and found that macrophages, microglia, pericytes, and oligodendrocyte precursor cells exhibited the most pronounced changes, with their m^1^A scores significantly decreased at all post-injury time points. Neutrophils, oligodendrocytes, and ependymal cells displayed an initial increase at 1 dpi, followed by a decrease at 3 dpi and 7 dpi. Dividing myeloid cells, endothelial cells, and astrocytes showed no significant changes during the acute phase but began to exhibit downregulation starting at 3 dpi. Dendritic cells and monocytes demonstrated a decrease only at 7 dpi. No significant changes were observed in fibroblasts or lymphocytes before and after injury (Fig 2F).

### 2.3. Dysregulation of transcriptional networks in myeloid cells and its close relationship with m^1^A score after SCI

**Fig 3:**
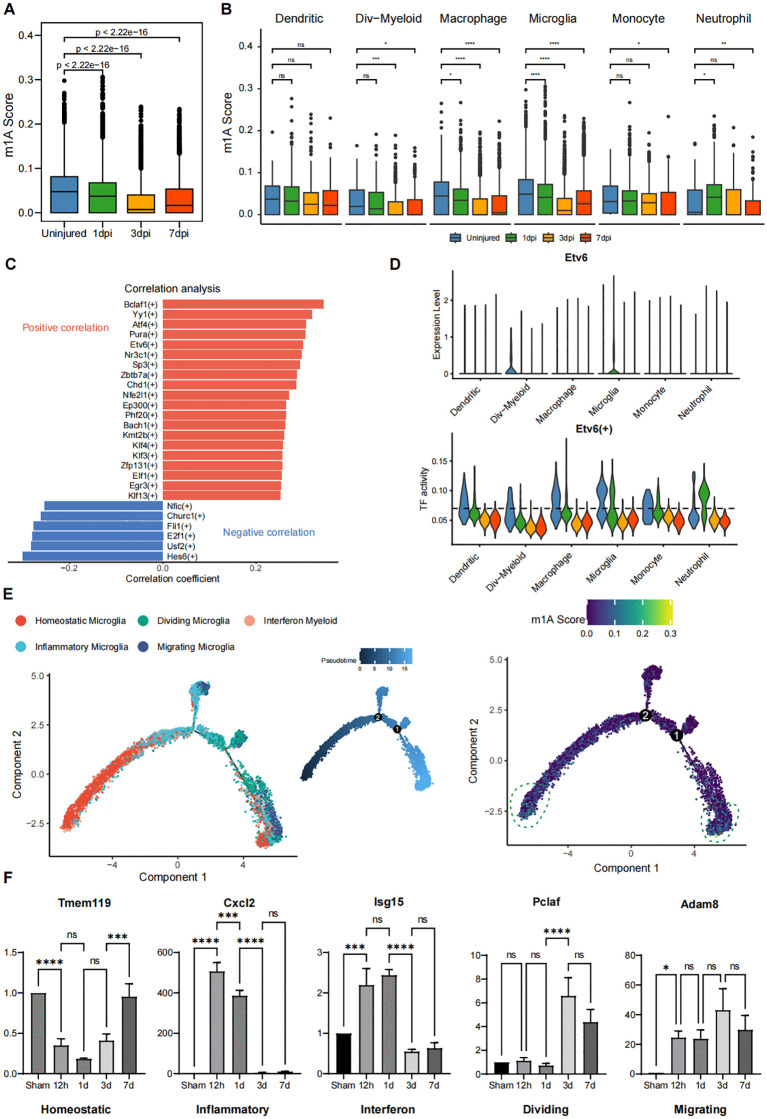
The dysregulated myeloid cell regulatory network after spinal cord injury is closely related to m^1^A modification. **(A, B)** Changes in m^1^A scores over time at the overall myeloid cell level and in various myeloid cell types. For all post-injury comparisons, the Uninjured group served as the baseline control, and score changes were interpreted relative to the Uninjured baseline. Uninjured: n = 3 mice; SCI: n = 2 mice. Each dot represents one cell. Significance levels are defined as follows: *P* < 0.05 (*), *P* < 0.01 (**), *P* < 0.001 (***), and *P* < 0.0001 (****); **(C)** Correlation analysis between m^1^A score and various transcription factors, with red indicating positive correlation and blue indicating negative correlation; **(D)** Expression changes of *Etv6* and transcription factor activity in different myeloid cell types at different time points after injury. Post-injury changes were evaluated relative to the Uninjured baseline; **(E)** Pseudotime analysis showing the overlap between microglial phenotypic transition and m^1^A score trends; (F) qPCR analysis showing expression changes of different microglial marker genes at various time points after injury. All post-injury expression changes were calculated and interpreted relative to the sham baseline. Sham: n = 3 mice; SCI: n = 3 mice. Significance levels are defined as follows: *P* < 0.05 (*), *P* < 0.01 (**), *P* < 0.001 (***), and *P* < 0.0001 (****).

Through the analysis of m^1^A score trends in different cell subgroups, we observed that m^1^A score changes in myeloid cells were particularly striking. There exists a complex interaction between myeloid cells, often mediated by transcriptional regulatory networks. To further explore the potential function of m^1^A modifications in myeloid cells, we performed analysis specifically for these cells. First, we performed dimensionality reduction and clustering of the myeloid cell subgroups and found a significant increase in the number of myeloid cells in the SCI group compared to the uninjured group, with microglia showing the most prominent increase (S1A Fig), which might be related to the activation of inflammatory responses. We used the pySCENIC pipeline to calculate transcription factor activity scores across all cell types, and variance decomposition showed the extent of SCI’s impact on myeloid cells. Upon re-clustering at the regulon level, we found that umapRAS_1 primarily encoded information related to cell types (S1B Fig). To eliminate the influence of nonlinear dimensionality reduction from UMAP, we also performed PCA, which still indicated that pcaRAS_1 encoded the main cell type information (S1B Fig). To explore changes in m^1^A score in myeloid cells at different time points post-SCI, we mapped the score onto UMAP plots at various time points, showing the m^1^A score in microglia exhibited a significant decrease despite an increase in cell numbers (S1C Fig). Further analysis of the overall m^1^A score in myeloid cells revealed a significant decrease at 1 dpi, which continued through to 7 dpi, with the most notable decrease at 3 dpi (Fig 3A). Analyzing each subpopulation separately to remove the effect of cell numbers showed a decrease in m^1^A score across all myeloid cell subgroups, with microglia exhibiting the most pronounced change (Fig 3B). Correlation analysis revealed a positive correlation between m^1^A score and the activity of most transcription factors, and these transcription factors’ target genes encoded m^1^A writer proteins (e.g., Etv6) (Fig 3C and S1D Fig). At 1 dpi, a lower m^1^A score was associated with reduced activity of positively correlated transcription factors, further decreasing the expression of m^1^A writer proteins, which may contribute to the observed m^1^A score patterns at later time points (Fig 3D). Furthermore, pseudotime analysis showed that the changes in m^1^A score across different cell types coincided with the transition of microglia from homeostatic to a proliferative state, suggesting that changes in the m^1^A score co-occur with microglial state transitions and are consistent with a potential role in these processes (Fig 3E). To confirm the phenotypic transition of microglia post-SCI, we performed quantitative PCR on mouse spinal cord samples before and after injury. We selected *Tmem119, Cxcl2, Pclaf, Adam8*, and *Isg15* as markers for homeostatic, inflammatory, dividing, migrating, and interferon microglia [17], respectively. PCR results from spinal cord samples at 12 hours, 1 day, 3 days, and 7 days post-SCI were consistent with the pseudotime analysis results: Homeostatic microglia predominated in uninjured mice. In the acute phase (12h and 1d post-injury), homeostatic microglia decreased, while inflammatory, interferon, and migrating microglia significantly increased. At 3 dpi, homeostatic microglia remained at low levels, migrating microglia remained high, and inflammatory and interferon microglia decreased while dividing microglia increased. At 7 dpi, homeostatic, inflammatory, and interferon microglia recovered to baseline levels, but migrating and dividing microglia remained elevated, albeit with a downward trend (Fig 3F). These results confirm the phenotypic transition of microglia during SCI, with a shift from homeostatic to inflammatory and interferon, then to dividing, and ultimately back to homeostatic, with migrating microglia maintaining high levels throughout.

### 2.4. Significant differences in m^1^A score in neurons After SCI

**Fig 4:**
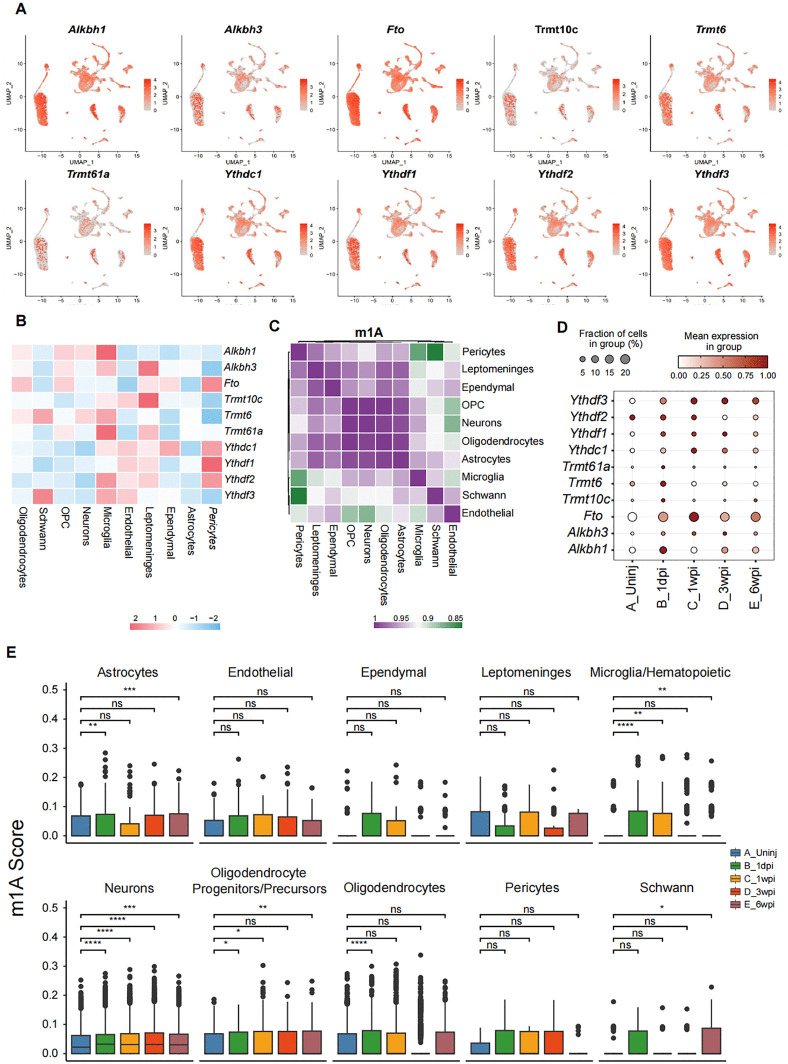
SnRNA-Seq sequencing analysis showing significant differences in m^1^A scores in neurons after spinal cord injury. **(A)** UMAP plot showing the expression of m^1^A regulatory factors across different cell types, with each dot representing a cell and color intensity representing m^1^A-related regulatory factor expression levels; **(B)** Heatmap showing the expression of m^1^A regulatory factors across different cell types, with color intensity representing expression levels; **(C)** Correlation of m^1^A-related regulatory factor expression levels across different cell subpopulations, with color intensity representing the degree of correlation (*r* < 0: negative correlation, *r* > 0: positive correlation); **(D)** Expression of m^1^A regulatory factors at different time points after spinal cord injury. For all post-injury comparisons, expression changes were evaluated relative to the Uninjured baseline; **(E)** Boxplot showing the m^1^A scores in different cell types at various injury time points. For all post-injury comparisons, the Uninjured group served as the baseline control, and score changes were interpreted relative to the Uninjured baseline. Uninjured: n = 3 mice; SCI: n = 3 mice. Each dot represents one nucleus. Significance levels are defined as follows: *P* < 0.05 (*), *P* < 0.01 (**), *P* < 0.001 (***), and *P* < 0.0001 (****).

Neurons are a critical cell type in SCI, and exploring the changes in m^1^A methylation modifications in neurons and different neuronal subtypes is essential for understanding the mechanisms of neuronal loss and dormancy after SCI. However, due to the relatively large soma size of neurons, conventional scRNA-seq often fails to capture them, making single-nucleus RNA sequencing (snRNA-seq) necessary to gather relevant information. Here, we utilized snRNA-seq data from SCI. To ensure alignment with the established cellular annotation, we referenced the UMAP spatial coordinates and cell classification from the original published studies during cell clustering and annotation (S2A Fig). Based on this, we further focused on the cell-specific expression of m^1^A modifications and found that the expression of m^1^A regulatory factors exhibited unique patterns across different cell types (Fig 4A and 4B). Heatmap analysis further revealed the specific expression of m^1^A regulatory factors across multiple cell groups (Fig 4C), consistent with the findings from bulk RNA-seq and single-cell sequencing. We then analyzed the changes in m^1^A score after SCI, finding that the expression of different m^1^A regulatory factors showed distinct trends, with the most significant increase in m^1^A regulatory factors on day 1 post-injury (Fig 4D). To further investigate the changes in m^1^A characteristics in different cell types post-SCI, we first performed UMAP clustering on sequencing results from different time points after injury (S2B Fig). Then, based on the overall expression of m^1^A factors, we constructed the m^1^A score (S2C Fig). Quantitative analysis of m^1^A scores in different cell types at different phases revealed significant differences in m^1^A score in spinal cord tissue compared to the uninjured, with neurons showing a significant escalating trend in m^1^A score at all time points post-injury, while other cell types exhibited less obvious differences (Fig 4E). These results indicate that neuronal transcriptional states are associated with higher m^1^A scores after SCI.

### 2.5. The m^1^A score is associated with neuronal energy metabolism after SCI

**Fig 5:**
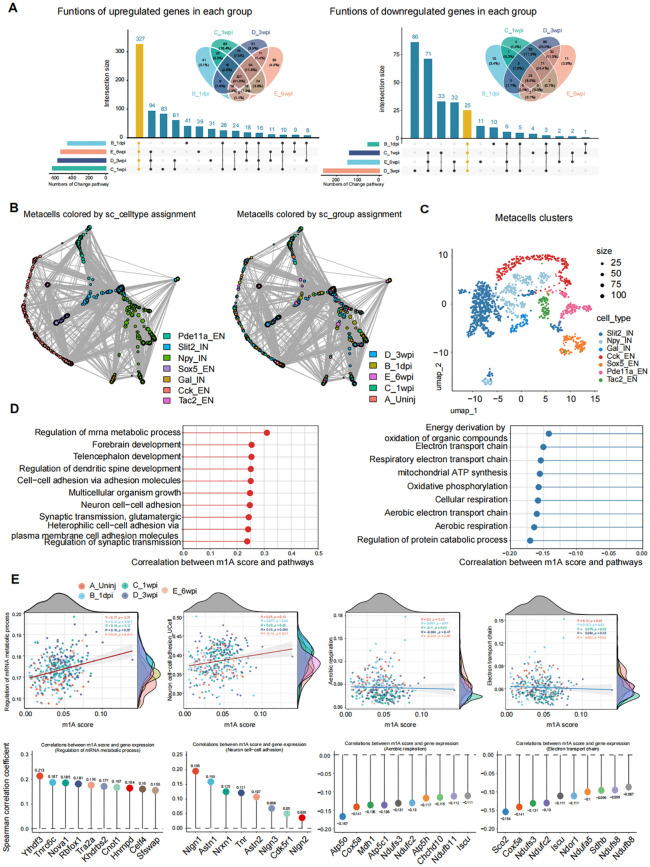
The m^1^A score is associated with neuronal energy metabolism after SCI. **(A)** Upset plot showing the overlap and divergence of enriched pathways derived from differentially expressed genes across multiple time points. The left panel displays the number of shared pathways among upregulated genes, and the right panel displays the number of shared pathways among downregulated genes. Venn diagrams indicate the intersection and respective proportions. Expressing changes strictly relative to the Uninjured baseline; **(B)** Force-directed distribution plot showing the distribution features of metacells, with the left plot showing neuronal subtype mapping defined at the single-cell level, and the right plot showing group mapping; **(C)** UMAP plot displaying the distribution of metacells and their sizes, with colors representing different neuron subtypes and sizes representing the number of cells within each metacell; **(D)** Lollipop plot showing the correlation between m^1^A scores and upregulated/downregulated functions in neurons, with red indicating positive correlation and blue indicating negative correlation; **(E)** Scatter plot showing the correlation between m^1^A score and positively/negatively correlated pathways at different time points, as well as the top 10 genes correlated with these pathways.

Since significant changes in m^1^A modifications were observed in neurons after SCI, we investigated whether these changes are linked to neuronal functions. We performed GO analysis on upregulated and downregulated genes in neurons at different phases post-SCI compared to the uninjured group, displaying the top five enriched pathways (S3A Fig). The activated pathways mainly included protein aggregation, histone modification, negative regulation of protein activity, and mRNA metabolism, while suppressed pathways were primarily related to oxidative phosphorylation, neuronal apoptosis, ATP metabolism, PI3K signaling, and neuron recognition functions (S3A Fig). To identify common pathways of change in neurons at different phases post-SCI, we used an Upset plot combined with a Venn diagram to display shared pathways across time points (Fig 5A). We found 327 common activated pathways and 25 suppressed pathways in neurons at different time points post-SCI (Fig 5A), suggesting that neurons remain in an activated state for an extended period after SCI. To further clarify the relationship between m^1^A score and specific pathways, we used SuperCell to aggregate the sparse single-cell matrix and performed force-directed clustering to display clustering results, mapping single-cell defined cell types and groups into constructed metacells. The results showed clear differentiation of cell types in the metacells, with no batch effects observed (Fig 5B). Subsequently, the integrated metacells were processed using the standard Seurat workflow analysis. After dimensionality reduction and clustering, the cell types defined at the single-cell level were mapped, similarly demonstrating the robustness of the cell type definitions (Fig 5C). After ensuring the reliability of the metacells, we calculated m^1^A scores and neuronal-related pathway scores at the metacell level using UCell, followed by correlation analysis. The results showed that m^1^A score was positively correlated with mRNA metabolism, neurodevelopment, and neuronal adhesion, and negatively correlated with oxidative phosphorylation, mitochondrial ATP synthesis, and aerobic respiration (Fig 5D). The correlation between pathway scores at different time points and m^1^A score was nearly identical, and further correlation analysis of genes in these pathways revealed the top 10 genes, which may be key molecules influenced by m^1^A score (Fig 5E).

### 2.6. Significant decrease in neuronal energy metabolism after spinal cord injury

**Fig 6:**
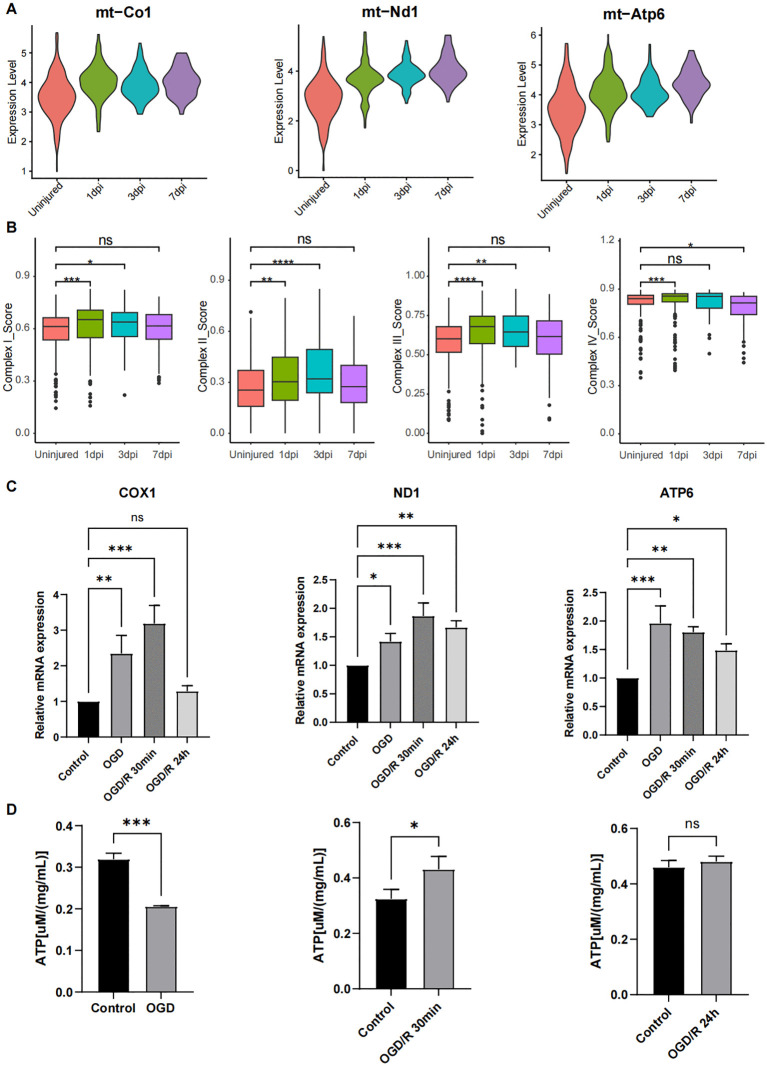
Neuronal energy metabolism significantly decreases after spinal cord injury. **(A)** Expression of various respiratory chain subunits at different injury time points. For all post-injury comparisons, expression changes were evaluated relative to the Uninjured baseline. Uninjured: n = 3 mice; SCI: n = 2 mice; **(B)** Expression of different respiratory chain complexes at various injury time points. For all post-injury comparisons, expression changes were evaluated relative to the Uninjured baseline. Uninjured: n = 3 mice; SCI: n = 2 mice; **(C)** HT22 cells were exposed to OGD for 12 h followed by reoxygenation for 0 min, 30 min, or 24 h to mimic ischemia–reperfusion injury, while control cells were left untreated. qPCR validation of respiratory chain complex I subunit ND1, complex IV subunit COX1, and complex V subunit ATP6 expression at different time points. Expression changes in OGD/R-treated cells were calculated and interpreted relative to the untreated control baseline. n = 3 per group; **(D)** ATP production levels at different time points. ATP levels in OGD/R-treated cells were compared with those in untreated control cells, which served as the baseline control. n = 3 per group. Significance levels are defined as follows: *P* < 0.05 (*), *P* < 0.01 (**), *P* < 0.001 (***), and *P* < 0.0001 (****).

Although bioinformatics analyses revealed a negative correlation between m^1^A score and neuronal energy metabolism after SCI, suggesting that neuronal energy metabolism decreases after SCI, this hypothesis had not yet been confirmed. To address this, we first analyzed the changes in respiratory chain complex subunits in neurons at the single-cell level after SCI. We found that nearly all respiratory chain subunits showed an increasing trend after SCI (Fig 6A, 6B and S4A Fig). We selected the most significantly changed subunits (*COX1, ND1*, and *ATP6*) for qPCR validation, and consistent with bioinformatics results, respiratory chain subunits were significantly upregulated in neurons after OGD (Fig 6C). After reoxygenation, expression of respiratory chain subunits further increased and peaked before declining. By 24 hours after reoxygenation, expression remained higher than in uninjured controls (Fig 6C). This contradicts the conventional understanding that energy metabolism is downregulated and respiratory chain subunits are damaged after injury. We speculate that reduced oxygen levels after SCI impair energy metabolism, while respiratory chain subunits are upregulated to counteract the hypoxic environment. To validate this hypothesis, we measured ATP levels at different time points post-injury. Considering that ATP levels change much faster than protein levels, we increased hypoxia severity and shortened hypoxia duration to preserve cell viability. The modeling conditions were changed to OGD 1h/R 0h, OGD 1h/R 0.5h, OGD 1h/R 24h. The results showed that ATP production significantly decreased after OGD but increased after 0.5 hours of reoxygenation, returning to baseline levels by 24 hours post-reoxygenation (Fig 6D). These results suggest that, post-SCI, tissue ischemia significantly reduces ATP production, while respiratory chain subunit expression increases as a compensatory response to hypoxia. Following reoxygenation, ATP production rapidly increases due to the higher expression and activity of respiratory chain subunits, and over time, both subunit expression and ATP production gradually return to normal levels.

## 3. Discussion

After SCI, the tissue undergoes a series of changes, including inflammation, astrocyte proliferation, neuronal degeneration, neural stem cell reactivation, demyelination, and cell death [42–44]. These changes contribute to a dynamic injury microenvironment post-SCI, which hampers the process of neural regeneration. Identifying the pathological and physiological changes of specific cells and their molecular mechanisms within this complex injury microenvironment is a challenging task. However, bulk RNA-seq and scRNA-seq provide promising insights into this issue, offering a way to study genetic expression changes at both the tissue and specific cell levels. Bulk RNA-seq analysis captures the overall gene expression changes in spinal cord tissue, while scRNA-seq identifies gene expression changes in specific cell types, thereby increasing the credibility of the results. However, it is essential to note that due to the cell size requirements for scRNA-seq, larger cells like neurons may not be adequately captured, leading to potential biases when analyzing neuronal cells using single-cell data [45,46]. To address this, we used snRNA-seq data to explore m^1^A methylation modifications in neurons post-SCI. Unlike scRNA-seq, snRNA-seq focuses on sequencing and analyzing individual cell nuclei, thus preserving the transcriptional profile of neurons to a greater extent, minimizing transcriptional bias, and improving the representation of cell types.

m^1^A methylation is a dynamic and reversible biological process involving methyltransferases (writers), demethylases (erasers), and methylation reader proteins. Writers catalyze the addition of a methyl group at the N1 position of adenosine in RNA, while erasers remove the m^1^A modification. Reader proteins recognize m^1^A-modified RNA and activate downstream regulatory pathways. Genes involved in m^1^A methylation are defined as m^1^A regulatory factors. Using GSVA and UCell methods, we have conducted a statistical analysis of the m^1^A scores for each group, representing the overall m^1^A regulatory factor activity in each cell [47,48]. The m^1^A score is an inferred proxy calculated from the expression of m^1^A regulator genes (writers/erasers/readers) using UCell/GSVA. It does not directly measure RNA m^1^A modification levels. The method of defining m^1^A score has been widely applied in studies of various physiological and disease mechanisms. For example, Zhao et al. used the m^1^A score to demonstrate that m^1^A modification is very important in the progression of hepatocellular carcinoma and the tumor microenvironment [49], while Gao et al. analyzed m^1^A score in oral squamous cell carcinoma cells to reveal the significance of m^1^A modifications for the prognosis of the disease [50]. However, such scores primarily reflect transcriptional programs related to m^1^A regulators and should be interpreted cautiously when discussing m^1^A modification levels. In this study, by utilizing m^1^A score, we explored the m^1^A modification in bulk RNA-seq, scRNA-seq, and snRNA-seq data before and after SCI and found that m^1^A score may exhibit time-specific and cell-type-specific characteristics in myeloid cell populations and neuronal subgroups after SCI.

In the bulk RNA-seq data, m^1^A regulatory factors and m^1^A score showed a significant increase on day 1 post-injury, with a decrease starting on day 3 and continuing through day 7. This indicates time-specific changes, suggesting that m^1^A may have distinct functional roles during different stages of SCI. Further analysis in scRNA-seq data focused on the changes of m^1^A score in different cell types, revealing a cell-type preference. Specifically, m^1^A regulatory factors showed a decrease in neutrophils, monocytes, and macrophages, while there was an increase in neurons, lymphocytes, astrocytes, and oligodendrocyte precursor cells, with the most significant changes observed in myeloid cells and neurons. Myeloid cells in spinal cord tissue include microglia and central-associated macrophages, which are part of the innate immune system of the central nervous system. After SCI, macrophages and microglia rapidly accumulate at the injury site, participate in inflammation, clear cell debris, and secrete various growth factors and cytokines to promote neuronal regeneration and repair [51,52]. Neurons, as key components of the nervous tissue, undergo necrosis, apoptosis, axonal breakage, and changes in neurotransmitter and receptor systems post-SCI [44,53].

Among the various cell types, myeloid cells exhibited the most significant changes in m^1^A score post-SCI. Analysis of scRNA-seq data before and after SCI showed that the m^1^A score decreased in all myeloid cell subgroups after injury, with microglia showing the most pronounced changes. In fact, methylation modifications in microglia are not uncommon. For instance, Li et al. reported that m^6^A methylation modifications regulate brain inflammation in rat microglia [54], and Ding et al. demonstrated that m^6^A methylation mediates microglial fate decisions [55]. Daily et al. showed that differential DNA methylation in microglia leads to *CASP4* overexpression, contributing to Alzheimer’s disease progression [56]. However, studies on m^1^A in microglia are sparse. In this study, we not only found a significant reduction in m^1^A score in microglia post-SCI but also identified through pseudotime analysis that m^1^A score may be linked to the phenotypic transition of microglia. Based on the classification of microglia into five subgroups by Lindsay [17], we confirmed that microglia undergo phenotpic transition after SCI: from homeostatic to inflammatory and interferon, then to dividing, and finally back to homeostatic, while migrating microglia remain high after injury without diminishing over time. The observed association between the m^1^A score and microglial state transitions suggests a testable hypothesis for future studies, including perturbation of specific m^1^A regulators to evaluate potential functional effects.

As the most important cell type, neurons play a crucial role in the repair of spinal cord injuries [57], and we found neurons also exhibit significant changes in m^1^A score post-SCI. RNA methylation modifications are abundant in neurons, and numerous studies have confirmed that methylation influences neuronal functions. For example, Shi et al. [58] found that YTHDF1, a methylation reader protein in the hippocampal neurons of adult mice, promotes protein synthesis upon recognizing corresponding RNA methylation sites, thus enhancing learning and memory abilities. Kevin et al. [59] found that tRNA modifications (e.g., mcm5s2U and m^1^A) were significantly increased during non-associative learning, suggesting a functional link between non-coding RNA modifications and learning processes. Li et al. [60] found that compound B vitamins (VBco) could reduce neuronal damage by down-regulating the levels of DNA methyltransferases (DNMT1, DNMT3A, and DNMT3B) in hippocampal neurons and lowering mitochondrial methylation. In our snRNA-seq results, we observed a significant increase in m^1^A score in neurons post-SCI. GO enrichment analysis further revealed that m^1^A score was significantly negatively correlated with neuronal energy metabolism. Subsequently, we validated the changes in neuronal energy metabolism at different stages after SCI. Initially, during SCI, trauma and edema compress blood vessels, leading to local ischemia and decreased oxygen levels in the injury site. This inhibits oxidative phosphorylation and ATP production. To counteract hypoxia, respiratory chain complex subunits are upregulated. During reperfusion, the increase in oxygen levels causes a sudden rise in ATP production, surpassing the levels observed in uninjured controls. Over time, ATP production and subunit expression levels gradually return to normal. We speculate that mitochondrial dysfunction during ischemia-reperfusion injury may ultimately reduce ATP production below baseline levels, though this remains to be experimentally verified.

In terms of therapeutic implications, there are currently no studies directly demonstrating that targeting m¹A regulation can promote spinal cord injury (SCI) repair. Nevertheless, emerging evidence indicates that m¹A modification may influence the biological functions of various neural cell types, suggesting a possible—though still preliminary—relevance to neurological disorders. (i) Neurons**:** Transcriptome-wide profiling in cortical neurons subjected to oxygen–glucose deprivation/reoxygenation (OGD/R) showed that m¹A modification patterns are significantly altered under ischemia-like stress conditions. These changes were enriched in transcripts associated with synaptic signaling and neuronal survival pathways, suggesting that m¹A may be involved in post-transcriptional responses to neuronal injury [61]. However, whether these alterations play a causal role in functional recovery remains unclear. (ii) Neural stem cells (NSCs): ALKBH3, a recognized m¹A demethylase, has been reported to regulate hippocampal neurogenesis by removing m¹A marks from specific target transcripts (e.g., *Mmp15*), thereby affecting their stability and translation. ALKBH3-mediated m¹A modulation was associated with cognitive function, highlighting a potential role for reversible m¹A regulation in neural development and repair [62]. Still, the broader relevance of these findings to injury contexts such as SCI requires further investigation. (iii) Other glial populations: In astrocytes, microglia, and oligodendrocytes, m⁶A modifications have been shown to regulate cell-specific functions [63–65]. In contrast, studies focusing on m¹A in these glial cell types remain limited, and its functional significance in these populations has yet to be clearly defined. Taken together, current evidence supports an association between m¹A methylation and neuronal stress responses or neurogenesis, whereas its roles in glial cells and in SCI remain largely unexplored. Given that m¹A modification is reversible and dynamically regulated by enzymes such as TRMT6/TRMT61A and ALKBH3, further mechanistic and interventional studies will be necessary to determine whether modulating m¹A-related pathways could have therapeutic relevance in SCI.

In this study, we employed both scRNA-seq and snRNA-seq for data analysis. scRNA-seq enables the characterization of m^1^A scores across diverse cell types but has limited efficiency in capturing neurons. In contrast, snRNA-seq compensates for this limitation. In snRNA-seq datasets, neurons and glial cells may benefit from reduced stress-related transcriptional bias; however, cytoplasmic transcripts—particularly those associated with immune and metabolic functions—may be underrepresented, especially in microglia and endothelial cells. Therefore, scRNA-seq and snRNA-seq should be considered complementary strategies rather than interchangeable approaches. Then we analyzed m^1^A score dynamics in different cell types at different phases after SCI, identifying microglia and neurons as the most significantly affected cell types. Through pseudotime analysis and gene enrichment analysis, we found that m^1^A score may be associated with microglial phenotypic transition and neuronal energy metabolism. We also used experimental methods to validate the phenotypic transition in microglia and energy metabolism in neurons post-SCI. However, there are still limitations in this study: In our in vitro experiments, we chose to use HT22 cells rather than primary neurons. While HT22 cells are widely used to study neuronal stress responses and RNA regulatory mechanisms, the extrapolation of our in vitro findings to spinal cord neurons should be made with caution. Future studies employing primary spinal neurons and in vivo SCI models will be necessary to confirm the relevance of these observations. In addition, although we observed parallel changes between m^1^A regulatory factors and microglial phenotypic transition as well as neuronal energy metabolism following spinal cord injury, we cannot yet determine whether these associations reflect a causal relationship. Specifically, we have not directly confirmed that microglial state transitions or neuronal metabolic alterations are mediated by m^1^A modification. Although we performed experimental validation, the models used in this study—including the in vivo SCI model and the in vitro OGD/R model—do not directly manipulate m^1^A pathways (e.g., through knockout or overexpression of specific m^1^A writers or erasers). Both SCI and OGD/R induce broad transcriptional and epigenetic changes, such as alterations in RNA abundance, methylation, acetylation, phosphorylation, and transcription factor activation. Any of these processes could potentially contribute to neuronal metabolic dysfunction and microglial phenotypic transition. Therefore, our current experimental results cannot fully establish that the observed functional changes are specifically attributable to m^1^A regulation. Further mechanistic studies involving direct modulation of m^1^A regulators will be required to clarify the causal role of m^1^A in these processes. In future research, we plan to precisely intervene in m^1^A levels using methyltransferases or demethylases to clarify their roles in microglial phenotypic transition and neuronal energy metabolism, providing reliable evidence for understanding the molecular mechanisms behind SCI recovery.

## 4. Methods

### 4.1. Ethics statement

The Institutional Animal Care and Use Committee (IACUC) of Qilu Hospital, Shandong University granted ethical approval for all animal procedures (Approval No. SDU-IACUC-25036).

### 4.2. Data collection and quality control

The scRNA-seq and snRNA-seq datasets used in this study were obtained from two previously published studies: “Single-cell analysis of the cellular heterogeneity and interactions in the injured mouse spinal cord,” and “Single-cell atlas of spinal cord injury in mice reveals a pro-regenerative signature in spinocerebellar neurons”. The sequencing data is available in the NCBI GEO database (NO. GSE162610 and NO. GSE172167). Data were processed and analyzed by R package Seurat. The expression matrices and sample information from the above-mentioned single-cell sequencing data were imported into RStudio to create Seurat objects. Cells with fewer than 800 detected genes or genes detected in fewer than five cells were filtered out. All quality control steps were based on the standards provided in the original publications.

### 4.3. Data processing

Since the data used in this study comes from multiple studies, each study was analyzed separately to ensure the reliability of the conclusions. Each study’s samples may originate from different batches, and variations in operational conditions and machine states across different batches could influence sequencing results. To ensure consistent clustering of data from different samples, we used Seurat’s FindIntegrationAnchors and IntegrateData functions to integrate the samples. In the integrated analysis, mitochondrial effects were regressed out using the vars.to.regress function in ScaleData. The top 2000 most variable genes were calculated using the vst method, and the data were scaled based on these genes to allow comparisons across different cells. Given that single-cell sequencing generates high-dimensional data that is difficult to directly visualize, dimensionality reduction was performed to identify distinct cell populations. Data were normalized and subjected to Principal Component Analysis (PCA) based on the top 2000 highly variable genes. Normalization allowed for better comparability between samples, and PCA helped identify the principal components with the greatest variance, revealing clustering patterns of cell types and states. Afterward, the integrated data were reduced in dimensionality using Uniform Manifold Approximation and Projection (UMAP) to further cluster and group cells. The results were visualized using DimPlot and other methods.

### 4.4. Cell annotation

Cell annotations were consistent with those used in the original studies. The following cell markers were used: microglia/hematopoietic cells (*C1qa, Ctss, Gpnmb, Lgals3, Itgax, Ms4a4b, Cd3g, Nkg7*), homeostatic microglia (*P2ry12, Siglech, Tmem119*), inflammatory microglia (low *P2ry12*, high *Igf1*), astrocytes (*Slc7a10, Agt, Gfap, Vim*), macrophages (*Ms4a7, Pf4, Fabp4, Gpnmb*), endothelial cells (*Bsg, Cldn5*), ependymal cells (*Nnat, Dnah12*), neurons (*Snhg11, Rbfox1, Rbfox2, Snap25*), pericytes (*Vtn, Pdgfrb*), neutrophils (*S100a9, Mmp9, Ly6g, Cd177, Ltf*), monocytes (*Ccr2, F10, Plac8*), dividing myeloid cells (*Mki67, Cdk1, Ccnb2, Top2a*), fibroblasts (*Col6a1, Dcn, Lum, Col1a1*), oligodendrocyte precursor cells (*Cspg5, Tnr, Fyn, Tcf7l2*), oligodendrocytes (*Plp1, Mag, Mog*), mature oligodendrocytes/myelin (*Plp1, Mag, Mog*), Schwann cells (*Mpz, Pmp22*), and leptomeningeal cells (*Dcn, Col1a1*).

### 4.5. Differential expression analysis

To identify differentially expressed genes (DEGs) in neurons from different injury groups, FindMarkers was used. To ensure accuracy, only genes detected in more than 10% of neurons were included in the analysis. Differential genes were compared between neurons from the healthy control or sham surgery group and neurons from the SCI group. Genes with an adjusted *P* < 0.05 and Log2FC ≥ 0.25 were considered significantly different.

### 4.6. m^1^A Score Calculation

To quantify the m^1^A modification pattern in the single-cell sequencing dataset, an m^1^A score system was established. This scoring system takes into account changes in multiple m^1^A regulatory factors. All m^1^A regulators were treated as a gene set, and UCell was used to score this gene set in all cells. Specifically, the full set of m^1^A regulators was defined as a cohesive gene module, and scoring was performed via the AddModuleScore_UCell function. The calculation was executed with a negative weight parameter (w_neg = 1) to balance the scoring, while storeRanks was set to FALSE to optimize memory efficiency during the ranking process. The analysis was conducted using a single thread (ncores = 1). The resulting enrichment values were formally defined as the m^1^A score and utilized for subsequent downstream comparative analyses across different cell states. In this study, the m^1^A score is an inferred proxy calculated from the expression of m^1^A regulator genes (writers/erasers/readers) using UCell/GSVA. It does not directly measure RNA m^1^A modification levels.

### 4.7. Functional annotation

DEGs underwent Gene Ontology (GO) and Kyoto Encyclopedia of Genes and Genomes (KEGG) pathway enrichment analyses. These analyses utilized the R package “clusterProfiler” (v4.4.0) [66]. The GO classification comprises three primary domains: Cellular Component (CC), Molecular Function (MF), and Biological Process (BP). The significance of gene enrichment for specific GO terms was assessed using adjusted *P*-values. For KEGG pathway analysis, genes were mapped to pathways to infer function. The enrichGO function within “clusterProfiler” was employed for GO analysis. Similarly, KEGG analysis was performed using enrichKEGG. Both GO terms and KEGG pathways exhibiting an adjusted *P* < 0.05 were deemed significantly enriched. For GSEA, all detected genes were pre-ranked based on their fold-change values, and enrichment scores were calculated against GO gene sets with 1,000 permutations. Terms with an adjusted *P* < 0.05 were considered statistically significant.

### 4.8. Transcription factor inference

Transcription factor (TF) activity within individual cells was investigated using the single-cell regulatory network inference and clustering (SCENIC) method to model gene regulatory networks across various cell types/clusters. SCENIC analysis was executed with the pySCENIC (0.12.1) package (utilizing GRNBoost2) following standard Python-based protocols [67]. The workflow encompasses three principal stages [68]: Co-expression Module Inference: GRNBoost2’s gradient boosting machine regression identified modules of co-expressed transcription factors and their potential target genes [69]. Module Pruning: Indirect target genes were removed by refining the modules using i-cisTarget software [70]. To estimate the regulatory activity of individual transcription factors (TFs), we employed the AUCell algorithm (v 1.32.0). For each identified regulon, an enrichment score (Area Under the Curve, AUC) was calculated based on the ranking of its target genes within the total expressed gene set of each cell. This process was executed with a minimum regulon size constraint (min.size = 10) to ensure statistical robustness, and the computation was parallelized using 10 cores. The resulting AUC values were utilized as a proxy for TF activity, allowing for the identification of state-specific regulatory drivers. Analysis relied on specific TF and motif annotation databases. The input was the normalized expression matrix derived from Seurat processing. TF activity scores were quantified as the AUC value calculated by AUCell for genes regulated by each TF.

### 4.9. Pseudotime analysis

Single-cell pseudotime trajectories were reconstructed using the Monocle2 (v 2.38.0) R package. To generate the CellDataSet object, the gene expression matrix, feature metadata, and phenotypic data from the GSE162610 dataset were integrated. Initial data filtering was performed to retain genes with a minimum expression threshold (min_expr) of 0.1 and a detection limit (lowerDetectionLimit) of 0.5. Following normalization, we identified differentially expressed genes to define cell progress. Dimensionality reduction was executed using the DDRTree (Discriminative Dimensionality Reduction Tree) algorithm to project cells into a lower-dimensional space. The microglial cell lineage was specifically extracted for manifold learning to characterize state transitions. Finally, we mapped the m^1^A scores onto the resulting trajectory to investigate the dynamic epigenetic alterations across microglial differentiation states.

### 4.10. Construction of SuperCell Metacells

Single-cell data are often noisy, and sparse data can lead to biased results when calculating gene and other feature correlations. To address this issue, we used SuperCell [71] to compute the similarity between single-cell matrices and performed KNN clustering, grouping transcriptionally similar cells into metacells, thus eliminating redundant information from individual cells while retaining biologically relevant heterogeneity. SuperCell metacells were constructed using the SCimplify function, with the normalized gene expression data matrix as input. PCA was performed to compute the KNN graph for metacell construction, and the following parameters were used: granularity level (gamma = 20), KNN number of adjacent genes (k_knn = 30), principal components used for KNN graph construction (nb_pc = 30), and number of most variable genes considered for PCA (nb_var_genes = 2000). The metacell gene expression matrix was generated via the supercell_GE function, where normalized expression values were aggregated by summation (mode = “sum”). To maintain biological interpretability, we performed high-resolution cell-type mapping using the supercell_assign function. Specifically, an absolute assignment method (method = “absolute”) was employed to map the original single-cell identity labels onto the resulting metacells, ensuring a high-fidelity representation of the initial cellular states. The refined metacell matrix was subsequently integrated into a Seurat object and processed following standard analytical pipelines for downstream exploration.

### 4.11. Animals

Female C57BL/6 mice (6–8 weeks) were sourced from GemPharmatech. (jiangsu, China). Mice were housed under a 12:12 hour light-dark cycle at regulated ambient temperatures, with free access to food and water.

### 4.12. Establishment of spinal cord injury model

Mice were induced into a state of anesthesia by using 4% isoflurane (rnbiology, R510, Shanxi, China) and anesthesia was maintained with 2% isoflurane. After T9 laminectomy to expose the spinal cord, a precise impact injury was delivered using an impactor (RWD, 68099II-S-M, Shenzhen, China) with the following settings: diameter, 1.0 mm; velocity, 1.2 m/s; dwell time, 0.8 s; penetration depth, 0.6 mm. Post-surgery, mice recovered consciousness on a warming cushion. Manual bladder expression was performed once a day post-operatively to aid urination.

### 4.13. Cell culture and OGD/R model

HT22 neuronal cells were obtained from Sunncell (Wuhan, China). Cultures were maintained in DMEM (Gibco, 11965092) supplemented with 10% FBS (Cellcool, CN1002L, Guangzhou, China) and 1% PS (Beijing Sunshine Biotechnology Co., Ltd., P1400, Beijing, China). To model SCI pathology in vitro, HT22 cells at 50–70% confluence were subjected to oxygen-glucose deprivation/reperfusion (OGD/R). This involved replacing the high-glucose DMEM with glucose-free DMEM (Gibco, 31053028) and incubating the cells under hypoxic conditions (1% O_2_, 5% CO_2_) for 12 hours. To simulate reperfusion, glucose-free DMEM was replaced with high-glucose DMEM at 0, 0.5, and 24 hours post-OGD (37°C, 5% CO_2_).

### 4.14. RNA extraction, cDNA synthesis, and qPCR

Tissue and cellular RNA were harvested using TRIZOL reagent. Total RNA extraction utilized the Total RNA Extraction Kit (Solarbio, R1200, Beijing, China). RNA quality was assessed via spectrophotometric A260/A280 ratio; acceptable ratios fell between 1.8 and 2.0. RNA was kept on ice prior to reverse transcription, which was performed using the RT reagent Kit (TaKaRa, RR047A, Beijing, China). For quantitative PCR (qPCR), cDNA was combined with TB Green Premix Ex Taq Kit reagents (TaKaRa, RR420A, Beijing, China) and specific primers (Table 1).

**Table 1:** The forward primers and reverse primers.

Gene Name	Forward primers	Reverse primers
β-Actin	GGCTGTATTCCCCTCCATCG	CCAGTTGGTAACAATGCCATGT
*Tmem119*	CCTACTCTGTGTCACTCCCG	CACGTACTGCCGGAAGAAATC
*Cxcl2*	CCAACCACCAGGCTACAGG	GCGTCACACTCAAGCTCTG
*Pclaf*	ACCAAAGCAAACTACGTTCCA	TTTTCCCGACGAACTTGAAGAA
*Adam8*	TTGCCCCATGTGAAACAGTATG	AGGTGCAGGGTGAAAACGTG
*I* *sg15*	GGTGTCCGTGACTAACTCCAT	TGGAAAGGGTAAGACCGTCCT
*ND1*	CCATTCTAATCGCCATAGCCTTCC	ATGCCGTATGGACCAACAATGTTAG
*COX1*	CCACTTCGCCATCATATTCGTAGG	TGTAAGCATCTGGGTAGTCTGAGTAG
*ATP6*	GCCCACCAACAGCTACCATTAC	TATAGGCTTACTAGGAGGGTGAATACG

### 4.15. ATP Measurement

After replacing high glucose DMEM with glucose-free DMEM, HT22 neuronal cells were placed under anoxic (0% O_2_, 5% CO_2_) conditions for 1 hour. Then, glucose-free DMEM was replaced with high glucose DMEM at 0, 0.5, and 24 hours (37°C, 5% CO_2_) to simulate reperfusion. ATP levels were measured using the ATP Assay Kit (Beyotime, S0026, Shanghai, China).

### 4.16. Statistical analysis

Statistical analyses were executed using GraphPad Prism 9 (GraphPad Software, San Diego, CA, USA) for experimental data and R (v.4.2.3) for high-throughput sequencing data. Statistical significance was defined as *P* < 0.05. Quantitative results are expressed as mean ± standard deviation, with significance levels denoted as *P < 0.05, **P < 0.01, ***P < 0.001, ****P < 0.0001, and ns indicating non-significant differences.

## Supporting information

S1 Fig The changes of m^1^A score in various myeloid cells and their correlations with transcription factors after spinal cord injury. (A) UMAP plot showing changes in 6 types of myeloid cells at different time points after spinal cord injury, with each dot representing a single cell; (B) Dimensionality reduction clustering of cell types and time points using different methods (left: cell expression clustering; middle: regulon activity UMAP; right: regulon activity PCA); (C) UMAP plot showing the distribution of m^1^A scores in myeloid cells at different time points; (D) Correlation analysis between m^1^A score and various transcription factors, with each dot representing a single cell.
(TIF)

S2 Fig The results of single-nucleus sequencing show the changes in m^1^A score in various types of cells. (A) UMAP plot showing the 10 major cell types in snRNA-Seq data from spinal cord tissue, with each dot representing a single cell; (B) UMAP plot showing the distribution of different cell types at various time points; (C) UMAP plot showing m^1^A score changes at different time points.
(TIF)

S3 Fig Enrichment analysis of differentially expressed genes in neurons after spinal cord injury. (A) Bubble plot showing the functions of upregulated and downregulated genes at different time points after spinal cord injury compared to uninjured samples, with bubble size representing the number of genes in the pathway and color representing -log10(p.adjust), with deeper red indicating smaller p.adjust values.
(TIF)

S4 Fig The changes of each respiratory chain subunit after spinal cord injury. (A) Expression of various respiratory chain subunits at different injury time points. For all post-injury comparisons, expression changes were evaluated and interpreted relative to the Uninjured baseline.
(TIF)
