# Supplementary figures and images for: Cell-type-specific m1A dynamics are associated with microglial phenotypic transition and neuronal metabolic adaptation during spinal cord injury
Source: PLoS Comput Biol. 2026 Jul 24;22(7):e1014573. doi: 10.1371/journal.pcbi.1014573 (PMC13423174; doi:10.1371/journal.pcbi.1014573)

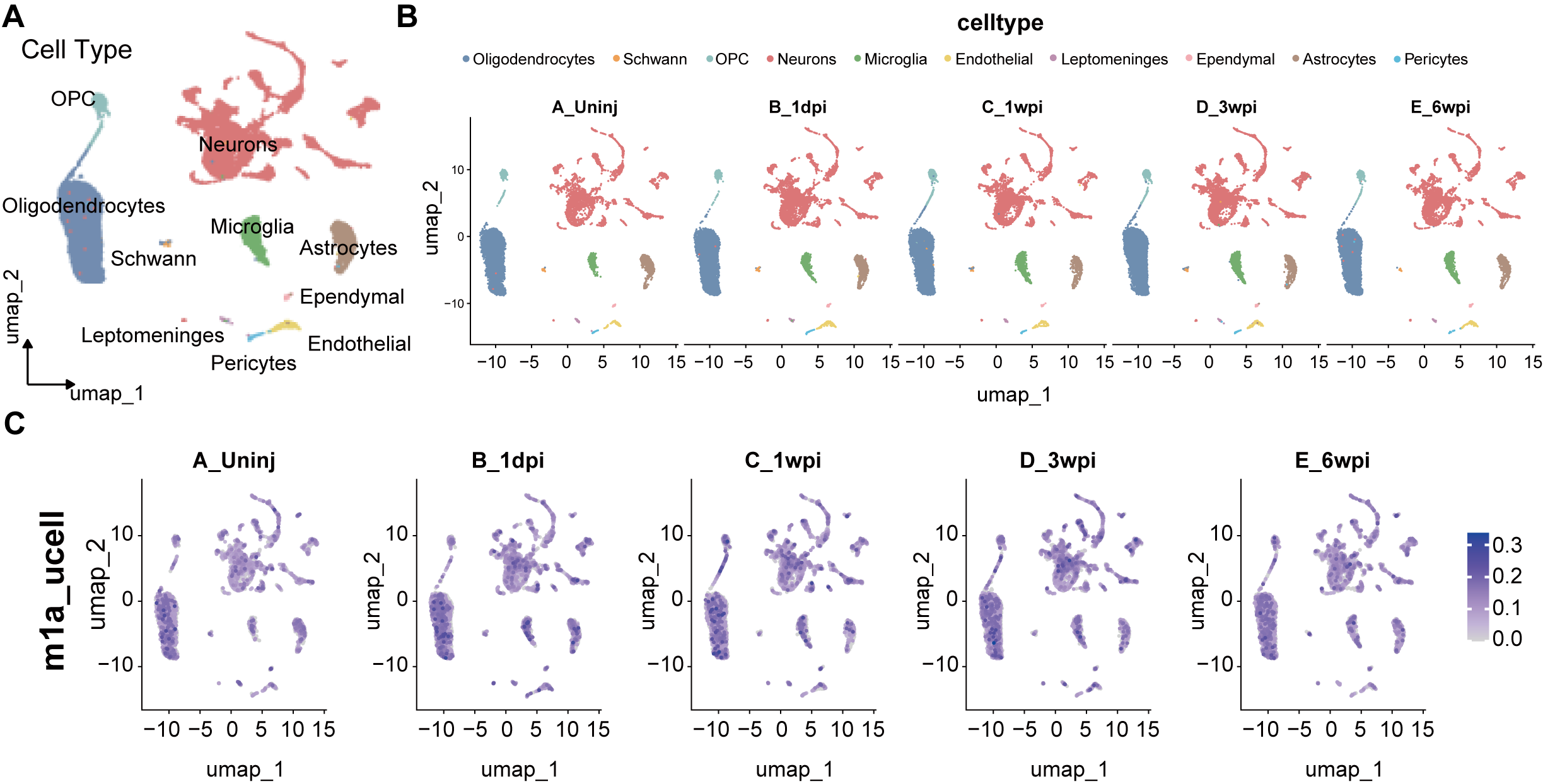

Supplement: S2 Fig — (A) UMAP plot showing the 10 major cell types in snRNA-Seq data from spinal cord tissue, with each dot representing a single cell; (B) UMAP plot showing the distribution of different cell types at various time points; (C) UMAP plot showing m1A score changes at different time points. (TIF) [file pcbi.1014573.s002.tif]
